# The large GTPase atlastin controls ER remodeling around a pathogen vacuole

**DOI:** 10.1080/19420889.2018.1440880

**Published:** 2018-03-06

**Authors:** Bernhard Steiner, Stephen Weber, Andres Kaech, Urs Ziegler, Hubert Hilbi

**Affiliations:** a Institute of Medical Microbiology, University of Zürich, Zürich, Switzerland; b Center for Microscopy and Image Analysis, University of Zürich, Zürich, Switzerland

**Keywords:** amoeba, atlastin, *Dictyostelium discoideum*, endoplasmic reticulum, dynamin-like GTPase, host-pathogen interaction, *Legionella pneumophila*, pathogen vacuole, type IV secretion

## Abstract

The ubiquitous environmental bacterium *Legionella pneumophila* is the causative agent of Legionnaires' pneumonia and replicates in free-living protozoa and mammalian macrophages in a specific compartment, the *Legionella*-containing vacuole (LCV). LCVs communicate with the endosomal, retrograde and secretory vesicle trafficking pathway, and eventually tightly interact with the endoplasmic reticulum (ER). In *Dictyostelium discoideum* amoebae and macrophages, the ER tubule-resident large GTPase Sey1/atlastin3 (Atl3) accumulates on LCVs and promotes LCV expansion and intracellular replication of *L. pneumophila*. Fluorescence microscopy of *D. discoideum* infected with *L. pneumophila* indicated that Sey1 is involved in extensive ER remodeling around LCVs. An ultrastructural analysis confirmed these findings. Moreover, dominant negative Sey1_K154A compromises ER accumulation on LCVs and causes an aberrant ER morphology in uninfected *D. discoideum* as well as in amoebae infected with avirulent *L. pneumophila* that lack a functional type IV secretion system. Thus, the large, dynamin-like GTPase Sey1/Atl3 controls circumferential ER remodeling during LCV maturation.

## Formation of an intracellular replication compartment by *L. pneumophila*

The causative agent of a severe pneumonia called Legionnaires' disease, *Legionella pneumophila*, is a facultative intracellular bacterium, which replicates in free-living protozoa and – after inhalation of bacteria-ridden aerosols – in lung macrophages [1]. An essential virulence factor of *L. pneumophila* determining the intracellular fate and pathogenesis is the bacterial Icm/Dot type IV secretion system (T4SS) [2]. The T4SS translocates more than 300 “effector” proteins into host cells, where they subvert signal transduction, as well as membrane and cytoskeleton dynamics [3,4]. The mechanism of intracellular replication is very similar in environmental and immune phagocytes, and thus, many Icm/Dot-translocated effectors target host proteins conserved in protozoan and metazoan organisms [5].

Dependent on the presence of the Icm/Dot T4SS, *L. pneumophila* forms within host cells a non-degradative, replication-permissive compartment termed the *Legionella*-containing vacuole (LCV). LCVs do not fuse with bactericidal lysosomes, but extensively interact with vesicles in the endosomal, retrograde and secretory trafficking pathways, and eventually associate with the endoplasmic reticulum (ER) [6–9]. While some evidence is available that the ER fuses with the LCV in murine bone marrow-derived macrophages (BMM) [10], fusion between the ER and LCVs is not observed in *D. discoideum* [5,11,12]. Rather, in the amoebae the LCV represents a distinct compartment, whose limiting membrane initially contains the phosphoinositide (PI) lipid phosphatidylinositol-3-phosphate (PtdIns(3)*P*), which is converted to PtdIns(4)*P* within 2 hours post infection [12,13]. Several T4SS-translocated effector proteins, including SidC and SidM, specifically bind PtdIns(4)*P* thus anchoring to the LCV membrane [14–21], and some effectors promote the recruitment of the ER to the pathogen vacuole.

### ER dynamics, atlastins and their role for pathogen vacuole formation

The ER is a highly dynamic and interconnected membrane system, consisting of tubular and sheet-like structures, which span the entire cell [22]. For proper cell homeostasis, ER membranes are constantly remodeled, and this process is mediated by a family of dynamin-like large GTPases, called atlastins [23]. Atlastins dimerize and upon GTP hydrolysis mediate the homotypic fusion of tubular ER membranes in various species: yeast Sey1p [24], plant RHD3 [25] or metazoan atlastin 1–3 (Atl1-3) [26,27]. The activity of atlastins can be compromised by mutating the phosphate-binding P-loop of the GTPase domain, as shown in human cells [26,28,29], *Saccharomyces cerevisiae* [24,26] and *Caenorhabditis elegans* [30]. A defect in GTP hydrolysis renders atlastins inactive, which in turn results in a disrupted ER morphology [31,32].

Recently, we identified Sey1/Atl3 in the proteome of LCVs isolated from *D. discoideum* and murine macrophages, respectively [33]. Using (live-cell) fluorescence microscopy, imaging flow cytometry and biochemical approaches, we demonstrated that *D. discoideum* Sey1 is indeed an atlastin orthologue, decorates ER-associated LCVs, promotes the expansion of pathogen vacuoles and ultimately supports the intracellular replication of *L. pneumophila* [13]. Specifically, we showed that the purified GTPase domain of *D. discoideum* Sey1 but not the K154A mutant hydrolyzed GTP, and thus, the catalytically inactive protein likely acts as a dominant-negative form by impairing GTPase activity or oligomerization of Sey1 dimers.

**Figure 1.**
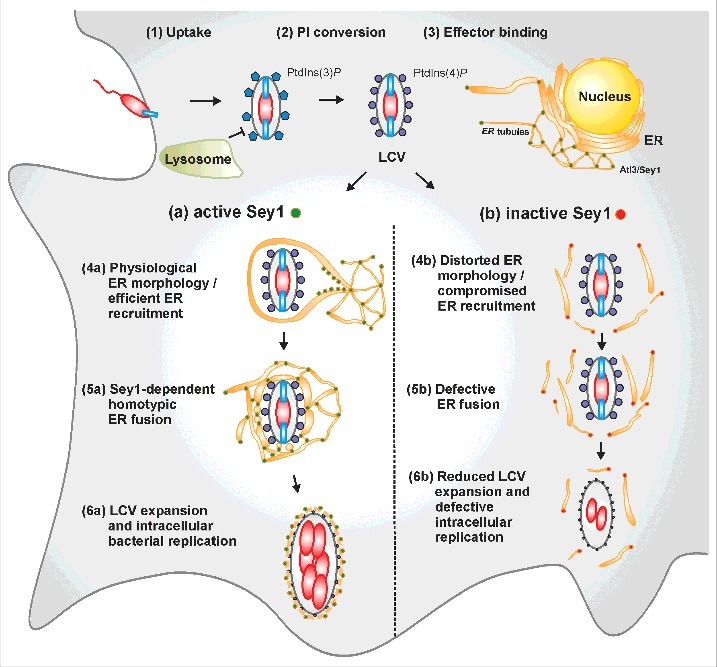
Schematic overview of Sey1/Atl3-dependent LCV formation. LCV formation is a sequential process, comprising the following steps: (1) uptake of *L. pneumophila* into a (phagocytic) host cell, (2) phagosomal phosphoinositide (PI) lipid conversion from PtdIns(3)*P* to PtdIns(4)*P*, and (3) binding of T4SS-secreted bacterial effector proteins (SidC, SidM) to PtdIns(4)*P* on the pathogen vacuole. These initial steps do not involve the ER tubule-resident large GTPase Sey1/Atl3. Active Sey1 subsequently promotes (4a) efficient ER accumulation on nascent LCVs, (5a) homotypic ER fusion around LCVs, and (6a) expansion of LCVs and intracellular replication of *L. pneumophila*. Production of the catalytically inactive, dominant negative mutant Sey1_K154A leads to (4b) distorted ER morphology and compromised ER recruitment to LCVs, (5b) defective ER fusion and dynamics, and (6b) reduced expansion of LCVs and impaired intracellular replication of *L. pneumophila*.

Whereas Sey1 overproduction stimulates intracellular growth of *L. pneumophila* in *D. discoideum*, the catalytically inactive Sey1_K154A mutant protein restricts replication of the pathogen, similar to the depletion of Atl3 by RNA interference (RNAi) in mammalian cells [13]. Furthermore, we found that the production of GFP-Sey1_K154A in *D. discoideum* impairs the efficient recruitment of ER to PtdIns(4)*P*-positive LCVs, and Sey1 promotes the expansion of PtdIns(4)*P*-positive pathogen vacuoles [13] (Fig. 1). Interestingly, addition of GTP (but not GDP or a non-hydrolysable GTP analogue) to purified, ER-positive LCVs caused a Sey1-dependent aggregation and size increase of the pathogen vacuoles. It is presently not clear, how Sey1/Atl3 contributes to the expansion of the PtdIns(4)*P*-positive limiting LCV membrane. Since the atlastin-dependent ER architecture is required for targeting membrane proteins to the nuclear membrane [34], putative atlastin-mediated ER-LCV contact sites might analogously be required for protein and lipid exchange. In summary, we showed that Sey1/Atl3-dependent ER remodeling contributes to LCV maturation and intracellular replication of *L. pneumophila* [13] (Fig. 1).

### Ultrastructural analysis of Sey1-dependent LCV morphology

**Figure 2.**
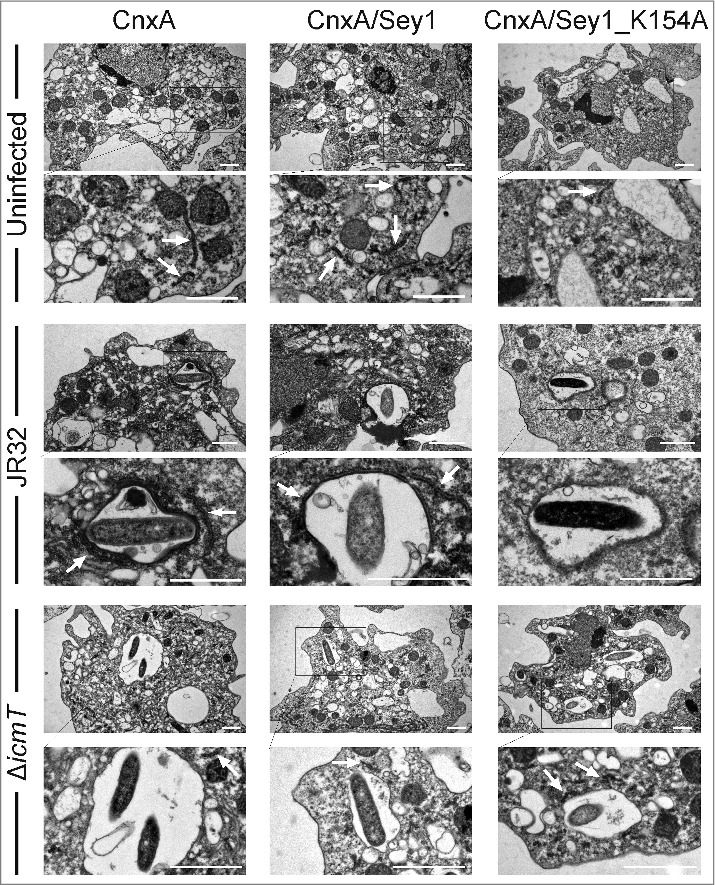
Transmission electron micrographs of *L. pneumophila*-infected *D. discoideum* producing Sey1 or Sey1_K154A. *D. discoideum* Ax3 ectopically producing calnexin-(CnxA) along with Sey1 or Sey1_K154A was infected (MOI 20, 2 h) with *L. pneumophila* JR32 or Δ*icmT*, chemically fixed and analyzed by transmission electron microscopy. Representative images are shown (scale bars, 1 µm). White arrows indicate rough ER. To allow parallel visualization by fluorescence microscopy, *D. discoideum* producing CnxA-mCherry in absence or presence of GFP-Sey1 or GFP-Sey1_K154A was used and infected with mCerulean-producing *L. pneumophila*.

To further investigate the role of Sey1 during LCV formation at an ultrastructural level, we performed electron microscopy (EM) with *D. discoideum* producing the resident ER marker calnexin-mCherry (CnxA-mCherry) alone or together with GFP-Sey1 or GFP-Sey1_K154A (Fig. 2). In uninfected amoebae, the production of CnxA-mCherry, or CnxA-mCherry concomitantly with GFP-Sey1, had no visible effect on the morphology of the cell, the structure of the ER, or the regions that displayed rough ER (Fig. 2, upper panels). Contrarily, the production of GFP-Sey1_K154A (together with CnxA-mCherry) in *D. discoideum* substantially reduced the occurrence of rough ER, leaving the cell almost devoid of the organelle. Endogenous Sey1 and ectopically produced, catalytically inactive GFP-Sey1_K154A likely form inactive mixed dimers, which might not hydrolyze GTP, do not assemble correctly or cannot complete the full membrane fusion reaction cycle.

LCVs harboring the virulent *L. pneumophila* strain JR32 in *D. discoideum* were decorated by rough ER preferentially when Sey1 was active [13] (Fig. 2, middle panels). Interestingly, upon production of GFP-Sey1_K154A no rough ER was detected around LCVs, even though CnxA-mCherry was ectopically produced in parallel. Phagosomes that contained an *L. pneumophila* T4SS-deficient mutant strain (Δ*icmT*) were completely devoid of ER membranes, regardless of whether GFP-Sey1 or GFP-Sey1_K154A was overproduced (Fig. 2, lower panels). In summary, the ultrastructural analysis by EM suggests that the adhesion of the ER to LCVs is a T4SS-specific process, which is promoted by host Sey1/Atl3 [13] (Fig. 1).

## Conclusions and outlook

We provided evidence that *D. discoideum* Sey1, like other large GTPases of the atlastin family, is a master regulator of ER morphology and dynamics in the amoebae. The production of catalytically inactive, dominant-negative GFP-Sey1_K154A suppresses the occurrence of rough ER membranes, which finally results in a less dense ER network. Newly transformed *D. discoideum* amoebae producing GFP-Sey1_K154A showed no observable defect in replication within the first two weeks of cultivation. However, after the third week in culture, we observed a distorted ER phenotype, with less-defined ER structures and a specific loss of rough ER membranes. Thus, at this point the amoebae appeared to have difficulties to cope with the dominant negative version of Sey1.

Intriguingly, *D. discoideum* producing GFP-Sey1_K154A was less permissive for intracellular *L. pneumophila*, and the LCVs did not expand as efficiently in these amoebae [13]. The ectopic production of dominant-negative GFP-Sey1_K154A (or depletion of Atl3 by RNAi) allowed us to study the involvement of a major cellular regulator of ER homeostasis during the bacterial infection cycle. For further analysis, the generation of a defined *D. discoideum Sey1* deletion mutant would be informative. However, given that only one Sey1/atlastin homologue is apparently present in the amoebae, the large GTPase might be essential. Future studies will address functional and mechanistic aspects about how Sey1/Atl3 regulates ER remodeling around LCVs, pathogen vacuole membrane expansion, nutrient availability, and intracellular growth of the major human respiratory pathogen, *L. pneumophila*.

## Abbreviations

Atl3: atlastin3
ER: endoplasmic reticulum
LCV: *Legionella*-containing vacuole
Icm/Dot: (Intracellular multiplication/Defective in organelle trafficking)
T4SS: type IV secretion system
Sey1: synthethic enhancer of YOP1 protein
